# Signatures of Antisymmetric Vibrations in the Ultrafast Dynamics of Quadrupolar Dyes

**DOI:** 10.1021/acs.jpclett.5c03526

**Published:** 2026-01-19

**Authors:** Somayeh Souri, Katrin Winte, Daniel C. Lünemann, Daniel Timmer, Teresa Kraus, Elena Mena-Osteritz, Peter Bäuerle, Sergei Tretiak, Christoph Lienau, Antonietta De Sio

**Affiliations:** † Institut für Physik, 11233 Carl von Ossietzky Universität, 26129 Oldenburg, Germany; ◧ Institut für Organische Chemie II und Neue Materialien, 9189 Universität Ulm, 89081 Ulm, Germany; § Theoretical Division and Center for Integrated Nanotechnologies, 5112 Los Alamos National Laboratory, Los Alamos, New Mexico 87545, United States; ∥ Center for Nanoscale Dynamics (CeNaD), 11233 Carl von Ossietzky Universität, 26129 Oldenburg, Germany

## Abstract

Antisymmetric molecular vibrations are central to ultrafast, nonadiabatic photophysical and photochemical processes such as conical intersection dynamics, Herzberg–Teller couplings and, potentially, singlet fission. Direct spectroscopic identification of such vibrations is, however, challenging, since they are typically Raman inactive and affect optical transitions only weakly. Here, we report experimental signatures of vibronic couplings to a high-frequency antisymmetric vibration in the excited state dynamics of a quasi-quadrupolar molecule by ultrafast two-dimensional electronic spectroscopy (2DES). The early time, sub-50 fs 2DES maps reveal an asymmetric peak pattern with characteristic low-energy cross-peaks. We show that these peaks arise from stimulated emission transitions from an anharmonic, double-minimum excited state potential energy surface formed by vibronic coupling to a high-frequency antisymmetric mode. Simulations based on a phenomenological essential state model support the results. Our findings offer a new approach for identifying antisymmetric vibrations in ultrafast 2DES and track excited state wavepacket motion before relaxation washes out the spectra.

## Graphical abstract

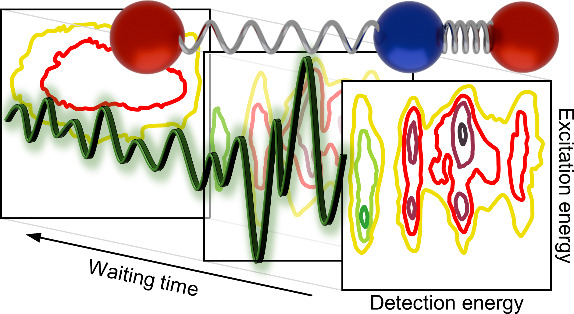

Quadrupolar molecules, based on acceptor–donor–acceptor (A-D-A) or donor–acceptor–donor (D-A-D) chromophores, are an important class of functional materials for photovoltaics,^1,2^ nonlinear optics,^3,4^ and bioimaging.^5^ They also emerge as model materials for studying light-induced nonadiabatic dynamics mediated by complex vibronic couplings.^6,7^ Phenomenologically, such dynamics may be understood by considering the molecule as composed of two identical polar donor–acceptor (D-A) complexes each coupling to an effective vibrational coordinate localized on the respective D–A arm.^6,8–10^ Relevant vibrations are typically high-frequency C–C stretching or ring breathing modes, common in conjugated molecules. Electronic coupling between the two D–A arms delocalizes not only the electronic wave function but also vibrations across the molecule, giving rise to symmetric *Q*_+_ and antisymmetric *Q*_–_ vibrational modes. The interplay of electronic coupling between the two D–A arms and vibronic couplings in each of the D–A arms is crucial for the photophysics of these chromophores. It leads to the emergence of an intramolecular charge transfer character^8^ in the electronic excitations of the molecule. These vibronic couplings and their effects on the optical spectra of quadrupolar molecules are often rationalized using phenomenological essential state models^6,8,10,11^ (ESM). For comparable electronic and vibronic coupling strengths, the ground state S_0_ is predicted to have small charge transfer character and to be well described by a harmonic potential, whereas the first excited state S_1_ results in a symmetry-broken double-minimum potential energy surface (PES) along *Q*_–_.^8^ Quadrupolar molecules falling in this regime are expected to undergo substantial fluorescence solvatochromism in polar solvents due to solvent-induced charge localization in the excited state.^6,8,9^ This is commonly known as excited state symmetry breaking in the literature.^12^ Recently, we have shown that excited state symmetry breaking of a prototypical quasi-quadrupolar A-D-A molecule (Figure 1a) in a polar solvent is more complex and involves two distinct microscopic processes:^13^ (i) the initial intramolecular vibronic dynamics and relaxation on the excited state double-minimum PES, and (ii) a subsequent solvent-induced symmetry breaking of the charge distribution resulting in charge localization on one D–A arm of the molecule as the relaxation process completes. The former process induces an ultrafast splitting and refocusing of the wavepacket, launched by optical excitation in the Franck–Condon region, across the two arms of the A–D–A complex. The latter leads to the commonly observed red-shifted fluorescence spectra of these quadrupolar molecules in polar solvents.

**Figure 1:**
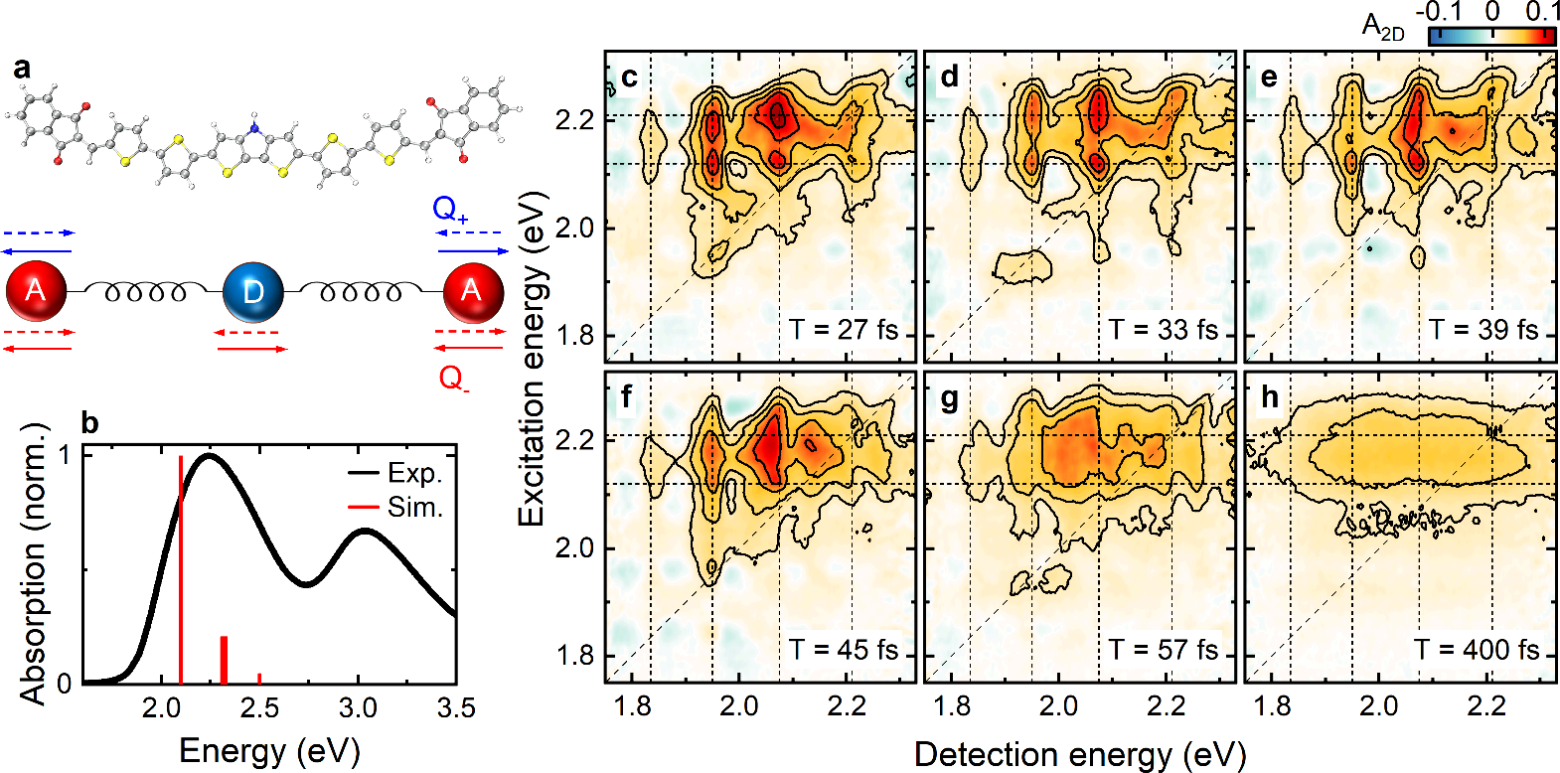
(a) Ball-and-stick representation of the investigated acceptor–donor–acceptor (A-D-A) molecule, composed of a central dithienopyrrole-thiophene donor unit linked to two symmetric indandione acceptor groups. Schematic visualization of the symmetric (*Q*_+_) and antisymmetric (*Q*_–_) modes in the essential state model (ESM). (b) Experimental (black) normalized linear absorption spectrum of the A-D-A molecule dissolved in cyclohexane together with the (red) stick spectrum obtained from ESM. The stick spectrum is normalized to the amplitude of the first stick. The *S*_0_ → *S*_1_ transition is at ∼2.1 eV. (c–h) Absorptive 2DES maps of the A-D-A molecule in cyclohexane at selected waiting times *T*. At early times, *T* < 50 fs (c–f), the maps show a vibronic peak pattern. This pattern and its dynamics reflect the impulsive excitation of coherent vibrational wavepacket motion on the excited state potential energy surface (PES). The peak pattern is asymmetric along the detection energy axis *E*_*D*_, with two peaks below and one peak above the main resonance at *E*_*D*_ ≈ 2.1 eV. This asymmetry is a direct consequence of vibronic coupling to *Q*_–_, inducing an anharmonic, double-minimum excited state PES.^13^ For waiting times beyond 50 fs (g and h), the vibronic peak pattern washes out, leaving a broad, featureless peak corresponding to the spectrum of the relaxed wavepacket.

Beyond excited state symmetry breaking, antisymmetric vibrations are key to the formation of conical intersections, governing many ultrafast photochemical reactions and nonadiabatic transitions.^14–18^ Moreover, they trigger dynamical symmetry breaking around Jahn–Teller distortions,^19^ modulate transition dipole moments via Herzberg–Teller couplings,^20^ are involved in phase transitions^21^ and potentially in singlet fission.^22,23^ As such, they play a fundamental role for photoinduced ultrafast dynamics. While the importance of antisymmetric vibrations has been well recognized and extensively explored in theoretical studies, their experimental detection remains challenging, since they are often Raman inactive. Compared to totally symmetric, typically Franck–Condon-active vibrations, the effect of antisymmetric modes on optical transitions is much weaker and mainly results in modulation of the electronic transition dipole moment along the vibrational coordinate.^20,24^ Quite generally, this made it difficult to directly probe the effects of antisymmetric modes on ultrafast optical spectra and the ensuing quantum dynamics.^25–29^

Here, we show clear experimental signatures of vibronic coupling to a ∼23 fs antisymmetric vibration in the excited state wavepacket dynamics of a quasi-quadrupolar A-D-A molecule in two-dimensional electronic spectroscopy (2DES). The early time, sub-50 fs 2DES maps reveal an asymmetric peak pattern arising from stimulated emission (SE) transitions enabled by vibronic coupling to the antisymmetric mode. This result demonstrates a path for experimentally accessing antisymmetric modes and probing their effects on quantum dynamics in solution before decoherence and relaxation set in.

For our study we use the quasi-quadrupolar molecule^13^ *2,2′-{{[4-(2-Hexyldecyl)-4H-dithieno­[3,2-b:2′,3′-d]­pyrrol-2,6-diyl]­bis­[3,4′-dihexyl-(2,2′-bithiophene)-5′,4-diyl]}­bis­(methaneylylidene)}­bis­(1H-inden-1,3­(2H)-dione)* as a model system. It consists of a central dithienopyrrole-thiophene donor unit linked to two indandione acceptors forming a symmetric, quasi-quadrupolar A-D-A structure^13^ (Figure 1a). To isolate the signatures of *Q*_–_ in 2DES, we perform all experiments with the molecule dissolved in nonpolar cyclohexane (CHX) to avoid solvent-induced symmetry breaking. The linear absorption spectrum of the A-D-A molecule in CHX (Figure 1b, black) shows a broad, stronger resonance at ∼2.1 eV arising from transitions between S_0_ and the vibronic manifold in S_1_. A second, weaker resonance at ∼3.0 eV is associated with transitions S_0_ → S_*n*_ (*n* > 2) to higher-lying electronic states.

Phenomenologically, the photophysics involving the ∼2.1 eV band can be reasonably well explained within a three-state ESM Hamiltonian^8^ comprising a charge neutral state (A-D-A) electronically coupled to two degenerate zwitterionic states (A^–^-D^+^-A and A-D^+^-A^–^). Recent work has shown that ESM provides a realistic framework for describing the photophysics of our molecule,^13^ suggesting that it can be parametrized by coupling each zwitterionic state to one high-frequency *ℏ*ω_*v*_ ≈ 1432 cm^–1^ bond stretching mode localized on one molecular arm. The dimensionless displacement of this mode, λ_*dia*_ = 0.75, corresponds to a Huang–Rhys factor of ≈0.3. The zwitterionic states are energetically separated from the neutral state by η = 2.1 eV and are electronically coupled to it with a coupling strength *t* = 150 meV. This coupling generates three effective PESs, the electronic ground state S_0_ and the first two excited states S_1_ and S_2_. These PESs are defined as a function of the effective vibrational modes *Q*_+_ and *Q*_–_, which are symmetric and antisymmetric superpositions^8^ of the stretching modes on each arm and are delocalized across the molecule. The comparable strength of electronic and vibronic coupling (*t* ≈ λ_*dia*_ *ℏ*ω_*v*_ ≪ η) in our molecule yields a double-minimum PES of S_1_ along *Q*_–_, whereas the PES is displaced by λ_*dia*_ along *Q*_+_.^13^

According to the ESM formalism, this puts our molecule in class I quadrupolar chromophores.^8^ Owing to the weak admixture of neutral and zwitterionic state in S_0_, these chromophores exhibit weak quadrupolar character of the ground state, defined by ρ = $0.5\left(1-\frac{\eta }{\sqrt{\eta^{2}+4t^{2}}}\right)$ . For our molecule, we estimated^13^ ρ ≈ 0.005. Additionally, the S_1_ and S_2_ are energetically close, with S_1_ being one-photon allowed and S_2_ two-photon allowed. While S_0_ and S_2_ are nonpolar, S_1_ becomes polar when the excitation dynamically localizes in one of the two equivalent minima,^8^ which ultimately may trigger solvent-induced charge localization in polar solvents. The double-minimum S_1_ PES is a fundamental characteristic of this class of A-D-A molecules, enabling excited state symmetry breaking.^13^ The details of the ESM Hamiltonian along with the relevant parameters are reported in the Supporting Information.

Ideally, the parameters of the ESM model would be derived directly from fully ab initio atomistic simulations. In practice, however, a unique assignment is not feasible. In the ESM framework, *Q*_+_ and *Q*_–_ coordinates are effective variables that do not correspond one-to-one to individual normal modes, but rather represent the combined contributions of multiple modes obtained from first-principles calculations. Atomistic simulations yield 3N–6 normal modes, differing slightly between ground and excited states,^13^ each with its own Franck–Condon and Herzberg–Teller activity. Visualization of the modes at relevant frequencies shows that they are predominantly symmetric or antisymmetric stretches.^13^ Projecting them onto the reduced ESM coordinate space is nontrivial and involves several approximations. Given this complexity, we choose for the present study to retain the ESM model in its original simplified form and fit the parameters directly to experimental spectra, which provides good agreement with the measurements.

The resulting stick absorption spectrum obtained from ESM (Figure 1b red) shows a dominant peak at ∼2.1 eV, the electronic S_0_ → S_1_ transition, and two weaker, quasi-degenerate peaks at ∼2.28 eV arising from transitions from S_0_ to energetically close-lying vibronic states in the S_1_ manifold. The absolute amplitude of the sticks is determined by the amplitude square of the transition dipole moment matrix elements μ_*j*,0_ = ⟨*j*|μ̂_*S*_|0⟩ between the ground state without vibrational excitation (|0⟩) and the j-th excited vibronic state in the S_1_ manifold (Table S1). Transitions to the S_2_ manifold are weaker (Table S1). This stick spectrum thus describes vibronic contributions to the main absorption resonance.

For 2DES, we excite and probe the low-energy resonance with a sequence of three broadband ∼9 fs pulses (Figure S1) in a partially collinear geometry. Experimental details are reported in the Supporting Information. Absorptive 2DES maps of the A-D-A molecule in CHX as a function of the excitation *E*_*X*_ and detection energy *E*_*D*_ at selected waiting times *T* are shown in Figure 1 and Figure S2. Reference 2DES measurements of pure CHX under similar conditions (Figure S3) show that cross-phase modulation^30^ from the electronic solvent response is only relevant for *T* < 20 fs. Even then, the characteristic dispersive line shape differs from the molecule’s 2DES signals and the peak amplitude is at least a factor of 2 weaker. All 2DES maps of the A-D-A molecule are therefore reported without solvent subtraction.

Up to *T* ≈ 50 fs, the 2DES maps of the A-D-A molecule show distinct, spectrally narrow vibronic peaks with two diagonal peaks at ∼2.1 eV and ∼2.2 eV and multiple cross-peaks. As we will argue below, this peak pattern reflects the impulsive excitation of a coherent wavepacket on the excited state PES (Figure 1c–f). The peak pattern appears asymmetric along *E*_*D*_ around the main 2.1 eV resonance, with one high energy and two low energy cross-peaks. For *T* > 50 fs (Figure 1g,h and Figure S2), the vibronic peak pattern washes out, leaving a broad, featureless peak at *E*_*X*_ ≈ 2.1 eV extending over the entire *E*_*D*_ axis. This reflects the spectrum after the relaxation of the excited state wavepacket.

We now analyze the early time 2DES peak structure to understand the origin of its asymmetry. Figure 2a shows an exemplary experimental 2DES map at *T* = 30 fs. The two rows of peaks at *E*_*X*_ ≈ 2.11 eV and *E*_*X*_ ≈ 2.21 eV (vertical inset) match reasonably well with the energy of the first two bright vibronic absorption transitions predicted by ESM (cf. Figure 1b red and Table S1). Along *E*_*D*_ we observe four peaks (horizontal inset), the main resonance at *E*_*D*_ ≈ 2.07 eV, a higher-energy peak at *E*_*D*_ ≈ 2.21 eV and two lower-energy ones at *E*_*D*_ ≈ 1.95 eV and ∼1.83 eV, respectively. This asymmetry along *E*_*D*_ is observed (Figure 1c–f) until the vibronic peak structure washes out. We also note that already at *T* = 30 fs, the main peak at *E*_*X*_ ≈ 2.10 eV, *E*_*D*_ ≈ 2.07 eV is slightly shifted off the diagonal (Figure 2a). We attribute this finite peak shift to fast solvent relaxation.

**Figure 2:**
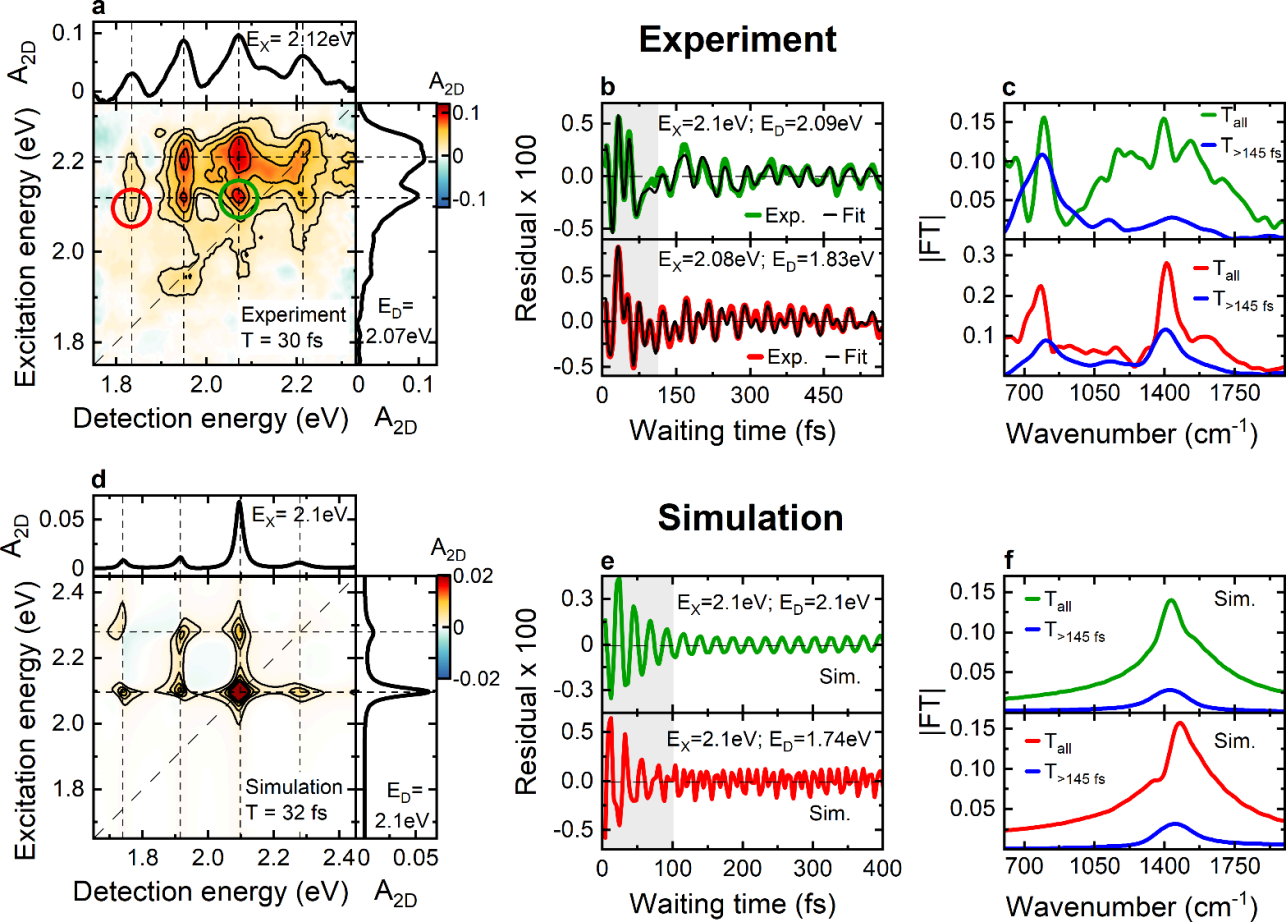
(a) Experimental absorptive 2DES map of the A-D-A molecule at *T* = 30 fs. Selected cross sections along the excitation axis, at *E*_*D*_ = 2.07 eV, and the detection axis, at *E*_*X*_ = 2.12 eV are shown in the insets. The peak pattern along *E*_*D*_ is asymmetric around the main resonance at ∼2.07 eV. (b) The amplitudes of selected peaks show ∼23 fs oscillations which rapidly decay within ∼100 fs, reflecting excited state wavepacket motion. Beyond *T* ≈ 100 fs, ground state wavepacket oscillations persist. Fits of the residual dynamics using a sum of damped cosine functions are shown in black. (c) Fourier transform (FT) spectra of the data in (b) for all *T* (green, red) and for *T* > 145 fs (blue) emphasize short-lived high frequency excited state oscillations around1430 cm^–1^ (23 fs). (d) Simulated absorptive 2DES map based on the parametrized potential energy surfaces (PES) derived from ESM at a selected *T* = 32 fs. Cross sections along *E*_*D*_ and *E*_*X*_ are reported in the insets. The peak pattern along *E*_*D*_ is asymmetric around 2.1 eV. (e) Rapidly damped oscillations of the peak amplitudes reflect excited state coherences, whereas the weaker longer-lived oscillations arise from wavepacket motion in the ground state. (f) FT spectra of the data in (e) for all *T* (green, red) and for *T* > 145 fs (blue).

The amplitudes of all the diagonal and cross-peaks display coherent oscillations along *T*. To emphasize them in Figure 2b, we remove the incoherent background amplitude. The resulting residuals are exemplary shown at the peaks *E*_*X*_ ≈ 2.10 eV, *E*_*D*_ ≈ 2.09 eV (green) and at *E*_*X*_ ≈ 2.08 eV, *E*_*D*_ ≈ 1.83 eV. (red). Oscillations appear on two distinct time scales. For *T* < 100 fs, rapidly decaying oscillations with a dominant period of ∼23 fs are observed. Beyond *T* ≈ 100 fs, weaker oscillations persist with a beating pattern indicating the excitation of an additional lower-frequency mode with ∼42 fs period. Fourier transforms (FT) of the data in Figure 2b for *T* > 145 fs (Figure 2c, blue line) reveal peaks at ∼1430 cm^–1^, confirming persistent ∼23 fs-period oscillations, and at ∼800 cm^–1^ (∼42 fs), indicating excitation of a solvent mode in the ground state of CHX.^31^

The A-D-A molecule has many intramolecular modes.^13^ Beyond high frequency modes around 1430 cm^–1^, the experimental Raman spectrum of the molecule^13^ shows also a much weaker, broad feature around 670–730 cm^–1^. Our recent DFT-based analysis also supports this observation^13^ indicating a mode around 700 cm^–1^ however with a Huang–Rhys factor three times smaller than that of the 1430 cm^–1^ modes. CHX features a strong Raman mode around 800 cm^–1^ (Figure S12). We thus attribute the strong, long-lived 800 cm^–1^ oscillations to the off-resonant excitation of a vibrational mode in the ground state of the solvent.

Reference transient absorption measurements of neat CHX (Figure S12a) show persistent oscillations with a dominant frequency of ∼800 cm^–1^. FT analysis of the temporal oscillations (Figure S12b) additionally reveals other three faint modes at 390 cm^–1^, 496 cm^–1^ and 1156 cm^–1^, consistent with Raman modes of CHX.^31^ Since these latter modes are significantly weaker than the 800 cm^–1^ one, it is unlikely that they influence the intramolecular dynamics under our experimental conditions.

We now compare the FTs in Figure 2c (blue) to those obtained from the data in Figure 2b for all waiting times (*T*_*all*_), including the initial 145 fs. The data at *E*_*X*_ ≈ 2.10 eV, *E*_*D*_ ≈ 2.09 eV (green) reveal impulsive excitation of rapidly damped oscillations around 1430 cm^–1^ (Figure 2c and Figure S4). The analysis evidence additional modes with decay times longer than 100 fs. The results are summarized in Table S3. Quantum chemical calculations^13^ show that several Raman-active modes of the conjugated backbone around 1400–1500 cm^–1^ contribute to the molecule’s linear optical spectra.

To rationalize these observations, we compare the experimental 2DES maps to simulations based on ESM.^8,13^ The resulting two-dimensional PES along *Q*_+_ and *Q*_–_ is schematically shown in Figure 3a. In these simulations, we take the system Hamiltonian in the form parametrized in ref.,^13^ without further parameter optimization. The electronic dephasing rate and the excited state vibrational relaxation rates are the only free parameters of the model. Vibrational relaxation rates of (50 fs)^−1^ are used for both *Q*_+_ and *Q*_–_, consistent with the experimentally observed rapid decay of the oscillation amplitude in the 2DES measurements (Figures 2 and S4) and further supported by our recent transient absorption studies of the same A-D-A molecule.^13^ The electronic dephasing rate is set to (50 fs)^−1^. The simulated 2DES maps (Figure 2d and Figure S5) show a well-resolved vibronic peak pattern with peaks along *E*_*X*_ at *E*_0_ = 2.1 eV and *E*_0_ + *ℏ*ω_*v*_ = 2.27 eV. As in the experiment, the peak pattern along *E*_*D*_ appears asymmetric around the main resonance *E*_0_, showing a pronounced cross-peak at *E*_0_ – 2*ℏ*ω_*v*_ = 1.74 eV. The simulated 2DES map is reasonably similar to that observed in the experiments. In contrast to the experiment, the simulations show asymmetry along *E*_*D*_ for all *T* (Figure S5) because the vibronic peak pattern does not wash out for *T* > 100 fs, since solvation of the molecule in CHX and other spectral diffusion processes are not considered. The peak amplitudes show temporal oscillations with a dominant period *T*_*v*_ = 2π/ω_*v*_ ≈ 23 fs, as depicted for two selected peaks in Figure 2e. As observed experimentally, a significant part of the amplitude oscillation decays within ∼100 fs, due to vibrational relaxation in the excited state. Weaker, long-lived oscillations persist for all *T* since relaxation in the ground state is not included in the simulations. The rapid oscillation amplitude decay during *T* < 100 fs (Figure 2e shaded) is also evident by comparing the FT spectra taken over all *T* (Figure 2f, green and red) and those for *T* > 145 fs (Figure 2f, blue). Interestingly, the persistent ground state oscillations at the lowest energy cross-peak *E*_*X*_ = *E*_0_, *E*_*D*_ = *E*_0_–2*ℏ*ω_*v*_ show not only a modulation with period *T*_*v*_, but also one at *T*_*v*_/2 ≈ 11 fs, as evidenced from the presence of a peak at 2860 cm^–1^ in the FT in Figure S6b.

**Figure 3:**
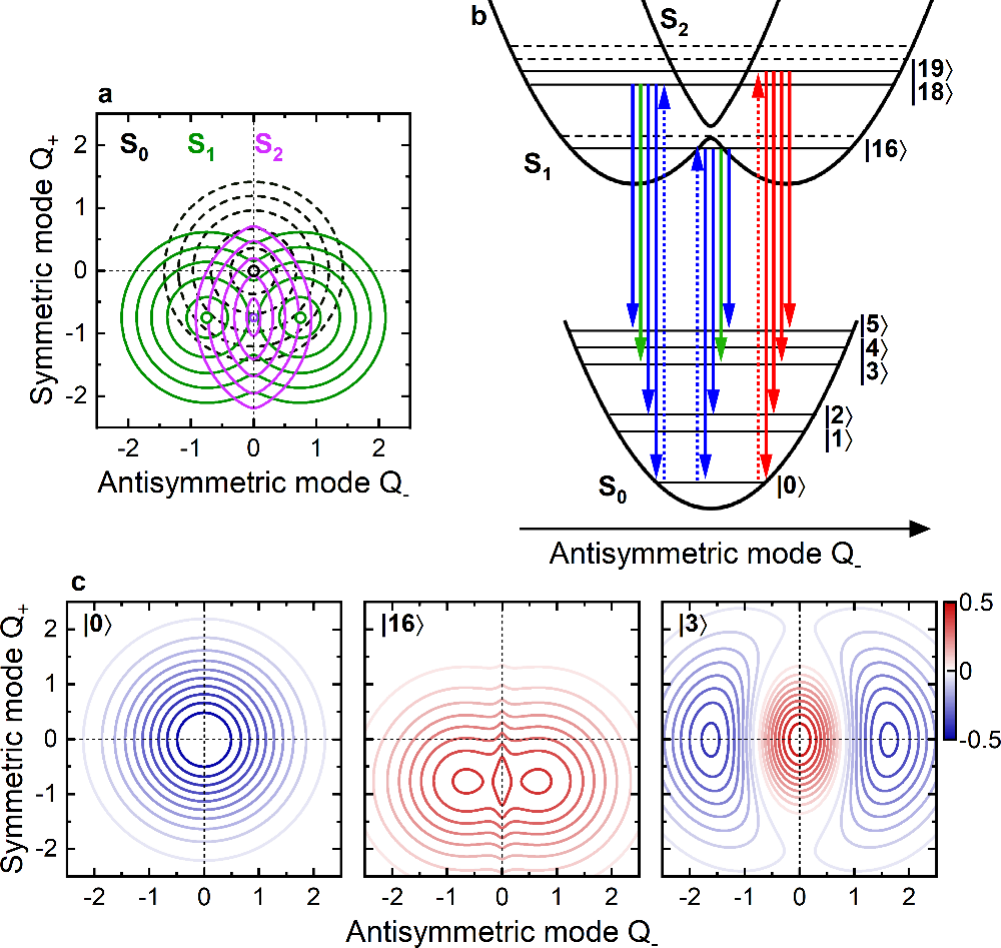
(a) Potential energy surfaces (PES) of the lowest-lying S_0_, S_1_ and S_2_ states along the symmetric *Q*_+_ and antisymmetric *Q*_–_ coordinates, as obtained by diagonalization of the ESM Hamiltonian. Vibronic coupling results in a symmetry-broken double-minimum PES in S_1_ along *Q*_–_ (green). (b) Schematic PESs (not to scale) along *Q*_–_ for a fixed *Q*_+_ = – 0.75 together with the lowest-lying vibronic energy levels in the three states. One-photon (two-photon) active states are denoted by solid (dashed) lines, respectively. Franck–Condon (FC) transitions are marked by blue, Herzberg–Teller (HT) transitions by red, and mixed FC/HT by green arrows, respectively. (c) Vibrational wave functions of three selected states involved in the transition |0⟩ → |16⟩ → |3⟩ that give rise to the new low energy cross-peak in the 2DES maps.

We note that the difference in relative peak intensities between simulations and experiment is a result of considering one Q_+_ and one Q_–_ mode in the ESM model. The experimental dynamics clearly indicate that additional Q_+_ modes are also coherently excited. Their contribution to the 2DES spectra results in symmetric peak patterns around E_0_ with energy spacings given by the specific Q_+_ mode frequency. Consequently, this will enhance the 2DES spectral intensity in particular for detection energies around *E*_0_ + ℏω_v_ and *E*_0_ – ℏω_v_. Because several of these additional Q_+_-type modes^13^ are close in frequency to 1430 cm^–1^, their combined contributions lead to increased intensities at these cross-peaks. Achieving closer quantitative agreement with the experiment would require higher-dimensional calculations explicitly including multiple vibrational modes.^32^

In general, the simulations reproduce reasonably well the most important experimental observations, namely the structure of the vibronic peak pattern at early *T*, its asymmetry along *E*_*D*_, and the rapid decay of the ∼23 fs peak amplitude oscillations within *T* < 100 fs. We therefore use these model calculations to qualitatively analyze the ultrafast quantum dynamics of the A-D-A molecule underlying the observed 2DES maps.

The effective PESs associated with S_0_, S_1_ and S_2_, given by the eigenvalues of the ESM Hamiltonian with fixed nuclear configuration (*Q*_–_,*Q*_+_), are shown in Figure 3a. Figure 3b reports a schematic cross-section of these PESs along *Q*_–_ for fixed *Q*_+_ = −0.75. It also depicts the relevant vibronic energy levels and transitions underlying the early time 2DES peak pattern.

In Figure 3b, we distinguish three classes of transitions between the ground and excited states. Franck–Condon-active (FC) transitions, which either keep all vibrational quanta unchanged (e.g., *|*0⟩ → *|*16⟩) or involve a change in the number of vibrational quanta along *Q*_+_, are depicted in blue. Herzberg–Teller (HT) transitions allow for a change of vibrational quanta along *Q*_–_ and are depicted in red or green. These HT transitions become allowed since the motion along *Q*_–_ alters the admixture of the two zwitterionic states in S_1_ and S_2_.^8,11^ This modulates the electronic transition dipole moment along *Q*_–_ making the transition HT-active.^20,24^ For SE, transitions are also relevant where the excitation occurs through a FC transition, whereas the SE back to the ground state involves a HT transition. Such transitions (Figure 3b, green) create a vibrational excitation with two vibrational quanta along *Q*_–_ in S_0_. We will discuss below that these FC/HT pathways contribute to the asymmetry of the 2DES peak pattern.

The early time 2DES vibronic peak pattern can be rationalized using the vibrational wave functions (Figure S10) associated with the system’s eigenstates. To rationalize the origin of the coherent peak amplitude oscillations observed along *T*, we need to understand how the impulsive optical excitation initiates coherent wavepacket motion on the excited and ground state PESs, how these wavepackets evolve, and how their signatures manifest in the 2DES maps.

The excited state wavepacket is launched on the double-minimum PES at the Franck–Condon point (*Q*_–_ = 0, *Q*_+_ = 0).^6,13^ Due to vibronic coupling, the wavepacket periodically oscillates along *Q*_+_ and splits along *Q*_–_ approaching the PES minima, before refocusing returning to the saddle point.^13^ This motion, characteristic of the molecule’s symmetry-broken excited state PES,^6,13^ thus consists of an oscillating component along *Q*_+_ and one along *Q*_–_, both with period *T*_*v*_ (Figure S8b). Its origin can be traced to the vibrational wave functions determining the coherent vibrational superposition in the excited state upon photoexcitation. The impulsive optical excitation induces transitions from |0⟩ into the excited state manifold, thereby creating a coherent superposition in the excited state mainly consisting of |16⟩, |18⟩, and |19⟩. The wave function associated with |16⟩ (Figure 3c) has no vibrational excitation. It shows a double-minimum shape along *Q*_–_ and is displaced along *Q*_+_ by −0.75. The wave function corresponding to |18⟩ reveals one vibrational excitation along *Q*_+_ and none along *Q*_–_ (Figure S10), whereas the one corresponding to |19⟩ has one vibrational excitation along *Q*_–_ and none along *Q*_+_. In a qualitative yet instructive picture, the relative amplitudes of the coefficients that weigh the eigenstates’ contributions in the coherent superposition can be estimated from the magnitudes of the transition dipole moments μ_0,*j*_, with *j* = 16, 18, 19 (Table S1), assuming impulsive excitation (flat spectrum across the relevant transitions). The coherent superposition of these three wave functions thus leads to an oscillatory motion along *Q*_–_ (|16⟩ and |19⟩) and another along *Q*_+_ (|16⟩ and |18⟩), both with period *T*_*v*_ (Figure S8b).

In 2DES, these two wavepacket motions are probed via SE pathways resulting in peak amplitude oscillations at early *T*. The emergence of the 2DES peak pattern can be rationalized in the framework of time-dependent perturbation theory.^33–35^ In particular, the lowest energy cross-peak (*E*_*X*_ = *E*_0_, *E*_*D*_ = *E*_0_–2*ℏ*ω_*v*_) is dominated by pathways in which the system is (i) initially excited into a coherence between the ground state |0⟩ and eigenstate |16⟩ by the first pump pulse. This coherence oscillates at *E*_0_ and thus contributes to peaks appearing at *E*_*X*_ = *E*_0_ in the 2DES maps. The interaction with the second pump pulse, (ii) brings the system into a coherent superposition of |16⟩ and |19⟩ which evolves during *T* oscillating with a period *T*_*v*_ as long as excited state wavepacket motion persists along *Q*_–_. The interaction with the probe pulse can then induce a transition from |19⟩ into |3⟩, (iii) leaving the system in a coherence between |16⟩ and |3⟩, thus oscillating at *E*_0_–2*ℏ*ω_*v*_, which subsequently radiates returning the system into the ground state in eigenstate |3⟩. This SE pathway (labeled *R*_*b*__1_ in Figure S11) thus results in a cross-peak at (i) *E*_*X*_ = *E*_0_ and (iii) *E*_*D*_ = *E*_0_–2*ℏ*ω_*v*_ with (ii) amplitude oscillating with *T*_*v*_. The wave function associated with |3⟩ has two vibrational excitations along *Q*_–_ (Figure 3c) and therefore the transition |16⟩ → |3⟩ is allowed by vibronic coupling to *Q*_–_. This explains the oscillating lowest-energy cross-peak observed in the 2DES maps (Figure 2a,b, red) and provides its dominant contribution (Figure S11, red frame). The emergence of this cross-peak thus primarily probes excited state wavepacket motion along *Q*_–_.

A much weaker oscillating contribution with period *T*_*v*_, probing excited state wavepacket motion along *Q*_+_, also appears at the same cross-peak position (*R*_*b*__3_ in Figure S11). It reflects a SE pathway in which the interaction with the second pump pulse induces a coherent superposition between |16⟩ and |18⟩. An oscillating cross-peak thus appears at the same energy position in the 2DES map and oscillates with period *T*_*v*_, but probes wavepacket motion along *Q*_+_. However, the contribution of this SE pathway is much weaker than that along *Q*_–_, since the relevant transition dipole moment μ_3,18_ < 0.1 μ_3,19_ (Table S2 and Figure S11). Another, weaker SE also arises from the interaction with the probe pulse bringing the system back to |5⟩ along *Q*_+_ (*R*_*b*__4_ in Figure S11), but it contributes only about half of that along *Q*_–_. In general, signatures of the motion along *Q*_+_ emerge in the 2DES map as essentially those of a displaced harmonic oscillator (DHO)^36,37^ with symmetric peaks along *E*_*D*_ around the main resonance (Figure S7). This alone, however, cannot explain the cross-peaks at *E*_*D*_ ≈*E*_0_–2*ℏ*ω_*v*_ observed in our experiment. The relevant SE pathways giving rise to this cross-peak are schematically summarized in Figure S11.

Analogously, the cross-peak at *E*_*X*_ = *E*_0_ + *ℏ*ω_*v*_, *E*_*D*_ = *E*_0_ – 2*ℏ*ω_*v*_ results predominantly from SE pathways along *Q*_–_. Specifically, the dominant contribution here is created by the interaction with the first pump pulse that excites the system into a coherence between |0⟩ and |19⟩. Subsequently, the second pump pulse brings it into a coherent superposition between |16⟩ and |19⟩ that oscillates with *T*_*v*_ during *T*, thus probing excited state wavepacket motion along *Q*_–_.

Excited state wavepacket motion persists until vibrational relaxation damps it into the two equivalent minima^13^ with a rate of ∼(50 fs)^−1^ in our molecule. After ∼100 fs, vibrational relaxation has substantially damped the excited state wavepacket (Figure S8b) and thus also SE oscillations rapidly decay on this time scale. This is seen as the initial, sub-100 fs decay of the peak amplitude oscillations in Figure 2b and 2e (gray shaded). As such, the appearance of an intense cross-peak at *E*_*D*_ = *E*_0_ – 2*ℏ*ω_*v*_ and its ∼23 fs oscillations decaying within ∼100 fs are signatures of excited state wavepacket motion along *Q*_–_.

Beyond ∼100 fs, much weaker peak amplitude oscillations persist (Figure 2b,e) which reflect underdamped vibrational wavepacket motion in the ground state of the molecule. A closer analysis of the ground state wavepacket suggests coherent motion only along *Q*_+_ (Figure S8a, blue), resembling essentially that described by a DHO model. Along *Q*_–_ in contrast, this wavepacket remains stationary at *Q*_–_ = 0 (Figure S8a, red) while its width “breathes” at *T*_*v*_/2 ≈ 11 fs (Figure S9b, orange). This behavior suggests that the impulsive optical excitation of the A-D-A molecule can induce a nonclassical vibrational wavepacket along *Q*_–_, resembling a squeezed state in the vibrational coordinate.^38^ This leads to a persistent overtone at 2*ℏ*ω_*v*_ (∼2860 cm^–1^, Figure S6b) and to high frequency beatings of the lowest-energy cross-peak’s dynamics for *T* > 100 fs in the simulations (Figure 2e, red). This overtone is, however, not resolved in the present 2DES experiment, possibly because the pulse duration (∼9 fs) is comparable with its oscillation period (∼11 fs).

Experimentally identifying antisymmetric modes in ultrafast spectroscopic measurements is usually challenging. Typically, impulsive resonant optical excitation launches coherent vibrational wavepacket motion in the excited and ground state driven by coupling of the electronic states to mainly totally symmetric, FC-active vibrations. In many cases, this coupling is well described by a simple PES shift along the coordinate, with the optical spectra then dominated by these *Q*_+_-type modes. The corresponding time-resolved nonlinear spectra are well understood within a DHO model.^39^ In contrast, vibronic coupling to *Q*_–_ modes usually results in minimal or no displacement of the PES and thus vanishingly small optical transitions, with different selection rules such as in Herzberg–Teller coupling.^20^

Our results reveal the effect of intramolecular vibronic coupling to a high frequency ∼1430 cm^–1^ antisymmetric mode in 2DES in a photoexcited quasi-quadrupolar molecule. Vibronic coupling to *Q*_–_ gives rise to an intrinsic double-minimum shape of the excited state S_1_ PES along *Q*_–_,^8,13^ which initiates intramolecular symmetry breaking in polar solvents.^13^ These features reveal that coupling to *Q*_–_ fundamentally alters the selection rules for optical transitions between electronic states, making transitions involving *Q*_–_ optically allowed that would instead be forbidden without vibronic coupling. In 2DES, the signatures of *Q*_–_ appear in the vibronic peak structure at early waiting times with the emergence of low-energy cross-peaks that oscillate with ∼23 fs period during the first ∼100 fs and make the pattern asymmetric along *E*_*D*_. These spectral signatures can be observed in the 2DES maps within the relaxation time of the excited state wavepacket (up to ∼50 fs in our molecule), before vibrational relaxation and rapid solvent relaxation wash out the vibronic peaks. We expect similar asymmetric 2DES peak structures to represent a general pattern for quadrupolar systems undergoing excited state symmetry breaking as for our A-D-A molecule, i.e., class I chromophores according to the ESM classification in which electronic coupling between the two molecular DA arms is comparable with vibronic coupling in each arm.^8^ Further 2DES investigations of other class I chromophores will be needed to establish the generality of this behavior.

In conclusion, we have shown that 2DES reveals distinct spectral and dynamical signatures of vibronic coupling to antisymmetric modes in a quasi-quadrupolar molecule undergoing excited-state symmetry breaking. The interplay between electronic and vibronic couplings in the A–D–A molecule, which forms a double-minimum excited-state PES, brightens transitions involving the antisymmetric mode that would otherwise be forbidden between purely harmonic PESs. Importantly, we can track the high-frequency oscillatory motion of the excited state wavepacket along the antisymmetric vibrational coordinate through the appearance of a distinct low-energy stimulated emission cross-peak in the 2DES maps. This cross-peak oscillates at the period of the antisymmetric vibration during the first ∼100 fs, directly tracking excited state wavepacket dynamics along *Q*_–_. Our results also show that, while the assumption of a single high-frequency mode underlying ESM is an approximation of the molecule’s complex multimode wavepacket dynamics, it provides a surprisingly accurate description of the most important spectral and coherent dynamical signatures of the coupling to the antisymmetric mode in 2DES. Our results open a new path for using ultrafast 2DES to identify the effects of antisymmetric molecular vibrations on the quantum dynamics of molecules in solution, thereby revealing the coherent dynamics governed by nonadiabatic couplings upon photoexcitation, before decoherence washes out the vibronic spectra. Overall, the experiments show that 2DES with subcycle vibrational resolution in the few-fs range represents a powerful method for interrogating the Hamiltonian of large molecules in solution, on time scales preceding intramolecular vibrational relaxation and solvation. Experiments with even higher temporal resolution than reported here may thus provide valuable new insight into electronic solvation and vibrational overtone dynamics in electronically excited states.

## Supplementary Material



## Notes

Sample preparation, experimental setup, model Hamiltonian and numerical simulations, and Figures S1–S13 (PDF)
