# Supplementary material for: Signatures of Antisymmetric Vibrations in the Ultrafast Dynamics of Quadrupolar Dyes
Source: J Phys Chem Lett. 2026 Jan 19;17(4):976–83. doi: 10.1021/acs.jpclett.5c03526 (PMC12862795; doi:10.1021/acs.jpclett.5c03526)
Supplement: Supplementary file 1 [file jz5c03526_si_001.pdf]

Supporting Information for

Signatures of Antisymmetric Vibrations in the Ultrafast Dynamics of Quadrupolar Dyes

Somayeh Souri[1,‡], Katrin Winte[1,‡], Daniel C. Lünemann[1], Daniel Timmer[1], Teresa Kraus[2], Elena Mena-Osteritz[2], Peter Bäuerle[2], Sergei Tretiak[3], Christoph Lienau[1,4], Antonietta De Sio[1,4]

1. Institut für Physik, Carl von Ossietzky Universität, 26129 Oldenburg, Germany

2. Institut für Organische Chemie II und Neue Materialien, Universität Ulm, 89081 Ulm, Germany

3. Theoretical Division and Center for Integrated Nanotechnologies, Los Alamos National Laboratory, 87545 NM, USA

4. Center for Nanoscale Dynamics (CeNaD), Carl von Ossietzky Universität, 26129 Oldenburg, Germany

‡ These authors contributed equally.

Correspondence to antonietta.de.sio@uni-oldenburg.de

Table of contents

1. Sample preparation 2

2. Experimental setup 2

3. Essential state model Hamiltonian and numerical simulations 3

4. Supplementary Figures 7

5. Supplementary Tables 14

6. References 16

1. Sample preparation

The quasi-quadrupolar molecule *2,2'-{{[4-(2-Hexyldecyl)-4H-dithieno[3,2-b:2',3'-d]pyrrol-2,6-diyl]bis[3,4'-dihexyl-(2,2'-bithiophene)-5',4-diyl]}bis(methaneylylidene)}bis(1H-inden-1,3(2H)-dione)* has been synthesized according to the procedure reported in Ref.[1]. The solutions used for the spectroscopic experiments presented in this manuscript are prepared by dissolving the molecule in cyclohexane (CHX) at a concentration of <1 mg/ml, which results in absorbance of 0.2 in a 1 mm path length.

2. Experimental setup

The quasi-quadrupolar molecule dissolved in CHX and pure CHX are measured using two-dimensional electronic spectroscopy (2DES) in a partially collinear geometry[2,3]. Broadband pulses with a spectral range of ~1.75 eV to ~2.32 eV (Figure S1a) are generated by a home-built non-collinear optical parametric amplifier (NOPA), pumped by a high-repetition rate Yb-based laser system (Carbide, Light Conversion) operated at 200 kHz. The pulses from the NOPA are compressed to 9 fs using a pair of chirped mirrors (DCM9 Laser Quantum). At the entrance of the 2DES setup, a beam splitter divides the NOPA pulses into the pump and probe arms. A phase-stable, collinear pulse pair is created in the pump arm using an in-line interferometer based on birefringent wedges (TWINS)[4,5]. The time delay τ (coherence time) between the two pump pulses is controlled by a motorized translation stage (M112.1DG1, Physik Instrumente). An additional chirped mirror pair (DCM9, Laser Quantum) is placed in the pump arm to compensate for the dispersion introduced by the TWINS. A small fraction of the pump beam is sent to a photodiode to record the autocorrelation of the two pump pulses on-the-fly during the measurements, which is used to calibrate the τ axis. The time delay between the second pump pulse and the probe pulse, the waiting time T, is controlled by a motorized translation stage (M126.DG, Physik Instrumente) in the probe arm. Both pump and probe beams are focused to a ~40 × 40 μm^2 spot size at the sample position using an off-axis parabolic mirror (OAP). The relative polarization between linearly polarized pump and probe pulses is 55°. The solutions of the A-D-A molecule in CHX and of the pure solvent are each filled into a 1 mm path length, fused silica cuvette (110-QS, Hellma).

To characterize the pulse duration at the sample position, a cross-correlation second-harmonic frequency-resolved optical gating (SH-FROG) measurement is performed between the pump and the probe pulses using a 10 μm thick beta barium borate (BBO) crystal (Figure S1). The resulting FROG map (Figure S1b) yields a retrieved pulse duration of about 9 fs.

After transmission through the sample, the probe pulses are dispersed in a grating spectrograph (Acton SP2150i, Princeton Instruments) and their spectrum is measured on a 2-shot basis using a high-speed line-camera (Octoplus, e2v) operating at 100 kHz. The pump beam is mechanically chopped at 50 kHz using a custom-built chopper system (MC2000B, Thorlabs) with a custom 500 slot wheel. The probe spectrum transmitted through the sample is recorded in the presence (I_{on}) and absence (I_{off}) of the pump. Differential transmission spectra $\frac{\Delta T}{T}(\tau, T, E_D) = \frac{I_{\text{on}}(\tau,T,E_D) - I_{\text{off}}(E_D)}{I_{\text{off}}(E_D)}$ are then obtained as a function of the time delays τ and T and of the probe energy E_D. To obtain absorptive 2DES maps, $A_{2D}(E_X, T, E_D)$, we take the real part of the Fourier transform of the $\frac{\Delta T}{T}(\tau, T, E_D)$ signal along τ. This yields absorptive energy-energy 2DES maps at each waiting time T, as a function of detection energy E_D and excitation energy E_X. The waiting time T is scanned in 3-fs steps from -45 fs to 600 fs and the coherence time τ is scanned from -83 fs to 196 fs. To improve the signal-to-noise ratio, 44 scans are recorded and averaged. All experiments are

conducted at room temperature. For all experiments, the pump fluence is set to 70 μJ/cm^2 and the probe to 55 μJ/cm^2. We have carefully ensured that these fluences are sufficiently low to keep the measurements in the regime of a third-order nonlinearity. Both sample absorbance and pump-fluence are kept as low as possible to ensure meaningful signals while maintaining a good signal-to-noise ratio.

3. Essential state model Hamiltonian and numerical simulations

Essential state models[6-10] have been used to describe the optical properties of the lowest lying excitations of quadrupolar A-D-A (or D-A-D) chromophores. We characterized the optical properties of our A-D-A molecule in a recent study[1] estimating the relevant parameters for the ESM from experimental linear absorption, photoluminescence, and pump-probe spectroscopy data, complemented by quantum chemical calculations. ESM considers the A-D-A molecule as composed of two coupled D-A dipoles, resulting in a neutral ground state $|N\rangle = |ADA\rangle$ and two zwitterionic states $|Z_1\rangle = |A^- D^+ A\rangle$ and $|Z_2\rangle = |AD^+A^-\rangle$. The zwitterionic states are separated from the neutral state by an energy gap η and they are electronically coupled to it with a coupling strength t. Vibronic couplings between electronic and nuclear degrees of freedom are introduced to account for the different nuclear configurations that are associated with the neutral and zwitterionic states[6]. Since charge redistribution from $|N\rangle$ to $|Z_1\rangle$ or $|Z_2\rangle$ occurs along the arms of the molecule, $|Z_1\rangle$ is coupled to a high-frequency vibrational mode localized on the left arm of the molecule, described by a dimensionless coordinate Q_1. $|Z_2\rangle$ is coupled to the same mode on the right arm, with coordinate Q_2. Vibronic coupling results in displacements λ_{dia} in the excited state potential energy surfaces (PESs) along these coordinates. The zwitterionic states are expressed as symmetric and antisymmetric combinations of $|Z_+\rangle = \frac{(|Z_1\rangle + |Z_2\rangle)}{\sqrt{2}}$ and $|Z_-\rangle = \frac{(|Z_1\rangle - |Z_2\rangle)}{\sqrt{2}}$. Analogously, symmetric $Q_+ = \frac{(Q_1+Q_2)}{\sqrt{2}}$ and antisymmetric $Q_- = \frac{(Q_1-Q_2)}{\sqrt{2}}$ vibrational coordinates delocalized across the A-D-A backbone are defined[6]. In terms of the $|N\rangle$,$|Z_+\rangle$ and $|Z_-\rangle$ states, the molecular Hamiltonian describing the free evolution of the system reads[9]

$$\begin{aligned} H_0 = \eta\{|Z_+\rangle\langle Z_+| + |Z_-\rangle\langle Z_-|\} + \sqrt{2}t\{|N\rangle\langle Z_+| + |Z_+\rangle\langle N|\} \\ + \hbar\omega_v\left(b_+^\dagger b_+ + b_-^\dagger b_-\right)|N\rangle\langle N| \\ + \hbar\omega_v\left[b_+^\dagger b_+ + b_-^\dagger b_- + \frac{\lambda_{dia}}{\sqrt{2}}\left(b_+^\dagger + b_+\right) + \lambda_{dia}{}^2\right]\{|Z_+\rangle\langle Z_+| \\ + |Z_-\rangle\langle Z_-|\} + \hbar\omega_v\left[\frac{\lambda_{dia}}{\sqrt{2}}\left(b_-^\dagger + b_-\right)\right]\{|Z_+\rangle\langle Z_-| + |Z_-\rangle\langle Z_+|\} \end{aligned} \quad (1)$$

where $b_+^\dagger$ (b_+) and $b_-^\dagger$ (b_-) are the ladder operators, namely creation (annihilation) operators, generating (destroying) one vibrational quantum in Q_+ and Q_-, respectively. In this basis, the effect of Q_+ appears as a regular displaced harmonic oscillator (DHO), whereas Q_- induces a linear coupling between $|Z_+\rangle$ and $|Z_-\rangle$. This vibronic coupling has a profound effect on the excited state PES of the molecule. We deduce the PES by numerical diagonalization of the Hamiltonian (eq. (1)) at each point in the effective coordinate plane with fixed nuclear configuration (Q_-,Q_+). We obtain the three eigenstates $|S_0\rangle$, $|S_1\rangle$ and $|S_2\rangle$ which describe the effective PESs of the A-D-A molecule in the electronic ground state (S_0) and in the two first excited electronic states (S_1 and S_2). When the strengths of electronic and vibronic couplings are comparable, as for our molecule, $|S_1\rangle$ shows an anharmonic PES with a double-minimum along Q_- and a displacement of $-\lambda_{dia}$

along Q_+. From our experiments, supported by quantum chemical calculations, we can estimate the relevant parameters as $\eta = 2.1$ eV, $t = 150$ meV, $\hbar\omega_v = 178$ meV and $\lambda_{dia} = 0.75$. The relevant dominant vibration is a C-C stretching mode with a frequency of 1430 cm^{-1} ($\hbar\omega_v \approx$ 178 meV)[1].

We describe the interaction of the system with light in semiclassical point dipole approximation. The interaction Hamiltonian in the basis of $|N\rangle, |Z_+\rangle$ and $|Z_-\rangle$ states is defined as follows

$$H_i(t) = -\mu_0 E(t)(|Z_+\rangle\langle Z_-| + |Z_-\rangle\langle Z_+|) \tag{2}$$

where μ_0 is the amplitude of the dipole moment of $|Z_+\rangle$ and $|Z_-\rangle$, and $E(t)$ is the external optical field exciting the system. For the 2DES simulations, this is a sequence of three time-delayed Gaussian pulses defined as $E(t) = \sum_k E_k(t)$ with $k = 1,2,3$ denoting the two pump pulses ($k = 1,2$) and the probe pulse ($k = 3$). Each optical field is taken as Gaussian pulse $E_k(t) = a_k e^{-2\ln 2\left(\frac{t-t_{0,k}}{t_w}\right)^2} \cos\left[\omega_0\left(t - t_{0,k}\right) + \phi_k\right]$, with carrier energy $\hbar\omega_0 = 2.2$ eV, pulse duration $t_w = 5$ fs defined as the FWHM of the field intensity profile, variable time delay $t_{0,k}$ with respect to the probe pulse ($k = 3$) which is centered at $t_{0,3} = 0$ fs, and phase ϕ_k. In the 2DES simulations, the phase ϕ_k is initially set to zero for each pulse and it is then cycled for the two phase-locked pump pulses to obtain the nonlinear signal as detailed below. For the simulations of the 2DES spectra, we numerically solve the master equation for the density matrix in the Lindblad form using a nonperturbative approach[11]

$$\frac{d\rho}{dt} = -\frac{i}{\hbar}[(H_0 + H_i), \rho] + \mathcal{L}(\rho) \tag{3}$$

Dissipation processes, such as vibrational relaxation and electronic dephasing, are accounted for phenomenologically in the Lindblad superoperator $\mathcal{L}(\rho) = \sum_m \gamma_m \left(2L_m\rho L_m^\dagger - L_m^\dagger L_m \rho - \rho L_m^\dagger L_m\right)$ where the rate constant γ_m describes the damping rate for the m-th damping process. Specifically, for vibrational relaxation, γ_+ describes the relaxation rate along Q_+ and γ_- the relaxation along Q_-, respectively. For these processes, the operators $L_+ = b_+$ and $L_- = b_-$ are the annihilation operators of the mode along Q_+ and Q_-, respectively. In these simulations we set the excited state vibrational relaxation time to 50 fs. This is chosen to be consistent with the experimentally observed relaxation of the excited state coherent vibrational wavepacket, as evidenced by the rapid decay of the oscillation amplitude in the 2DES measurements (Figures 2 and S4) and further supported by our recent transient absorption studies of the same A-D-A molecule[1]. In agreement with experiment, we assume the same 50 fs relaxation time for both Q_+ and Q_- coordinates in the excited state, i.e., we set $\gamma_+ = \gamma_- = (50 \text{ fs})^{-1}$. In contrast, our experimental results indicate that vibrational relaxation in the ground state is much slower, on the order of 1 ps or longer. As such, vibrational relaxation in the ground state is neglected over the time window of the present simulations. To obtain finite peak lineshapes in the 2DES spectra, we introduce electronic dephasing, destroying the coherence between the ground and excited states through the operators $L_d = |i\rangle\langle i|$ and we take the electronic dephasing rate as $\gamma_d = (50 \text{ fs})^{-1}$.

For the simulations, we initially define vibronic states $|n, k\rangle$. The quantum number $n = 1,2,3$ denotes the electronic states $|N\rangle$, $|Z_+\rangle$ and $|Z_-\rangle$, respectively, whereas $k = 0,1,\ldots,N_V - 1$ gives

the vibrational quantum number of the two-dimensional harmonic oscillator wavefunctions. Here, N_V is the total number of vibrational quanta considered for each electronic state. For the simulations presented in this work, we take $N_V = 16$ which results in 48 vibronic eigenstates, denoted $|j = (n-1)N_V + k\rangle$, with $j = 0, \dots, 47$. In Figure 3b of the manuscript, we indicate three relevant vibronic states involved in the optical transition giving rise to the low energy cross peak in the 2DES maps.

We emphasize that the electronic eigenstates, $|S_0\rangle$, $|S_1\rangle$ and $|S_2\rangle$, are superpositions of $|N\rangle$, $|Z_+\rangle$ and $|Z_-\rangle$ and are obtained by numerical diagonalization of the molecular Hamiltonian H_0 defined in eq. (1). Accordingly, the light-matter interaction, as well as the density matrix, in the basis of eigenstates can be obtained by applying a basis transformation to the ones defined above for the $|N\rangle$, $|Z_+\rangle$, $|Z_-\rangle$ basis. For the numerical simulations presented in the manuscript, we perform such a basis transformation and set the initial state before light-matter interaction takes place as $|S_0\rangle$ without any vibrational excitation, i.e., $|0\rangle$, which is the ground state of the system. Since the energy of the relevant vibrational mode ($\hbar\omega_v = 178$ meV) is much larger than the thermal energy at room temperature ($k_B T \approx 26$ meV), we perform the simulations by neglecting any initial thermal excitation of the system.

Transition dipole moment amplitudes obtained by diagonalizing the ESM dipole matrix at fixed nuclear configuration are reported in Table S1. We deduce Franck-Condon (FC) or Herzberg-Teller (HT) character of each transition between ground and excited state from the amplitude distribution of the involved wavefunctions in the (Q_-, Q_+) coordinate plane (Figure S10). Specifically, transitions that preserve all vibrational quanta or involve changes only along Q_+ are classified as FC, whereas transitions that involve changes in the number of vibrational quanta along Q_- are assigned to HT. The latter arise from coupling of the electronic states to Q_-, which alters the admixture of the two zwitterionic states in S_1 and S_2, thereby modulating the electronic transition dipole moment along Q_-, consistent with HT transitions.

Numerical solution of eq. (3) yields the time evolution of the density matrix of the system $\rho(\tau, T, t)$ as a function of the coherence time τ, the waiting time T and the simulation time t. The total polarization induced by the pulse sequence is obtained by computing the expectation value of the dipole operator. To extract the nonlinear, third-order signal corresponding to the phase-matching direction mimicking the one in the partially collinear configuration of the experiment, we apply a phase cycling algorithm[11,12]. For each τ and T, we calculate the total polarization for four different phases $\varphi_1 = \varphi_2 = n\,\pi/2$, with $n = 0,1,2,3$, between the pump pulse pair, in which E_1 and E_2 are phase-locked, and the probe pulse E_3, whose phase $\varphi_3 = 0$ is not cycled. After averaging these four contributions, we subtract the linear contributions to isolate the third-order nonlinear polarization $P^{(3)}(\tau, T, t)$. This contains quantum pathways contributing to the nonlinear response of the sample[13] which correspond to those probed in the partially collinear configuration of the experimental 2DES setup.

We also perform numerical simulations of 2DES dynamics using a two-mode DHO model as a reference. To this aim, in eq. (3) we use the following DHO Hamiltonian

$$H_0^{DHO} = \hbar\omega_v\left(b_+^\dagger b_+ + b_-^\dagger b_-\right)\left|S_0^{DHO}\right\rangle\left\langle S_0^{DHO}\right| + \left[\eta + \hbar\omega_v\left(b_+^\dagger b_+ + b_-^\dagger b_-\right) + \hbar\omega_v \frac{\lambda_{dia}}{\sqrt{2}}\left(b_+^\dagger + b_+\right) + \hbar\omega_v\lambda_{dia}^2\right]\left|S_1^{DHO}\right\rangle\left\langle S_1^{DHO}\right| \tag{4}$$

with the same values of η, $\hbar\omega_v$ and λ_{dia} as for ESM. At each position in the (Q_-, Q_+) coordinate plane, this Hamiltonian describes two two-dimensional harmonic oscillator PES with the excited state S_1^{DHO} PES displaced by $-\lambda_{dia}$ along Q_+ with respect to the ground state S_0^{DHO} PES. Both PESs remain undisplaced along Q_- . The light-matter interaction is governed by

$$H_i^{DHO}(t) = -\mu_0 E(t)\left(\left|S_1^{DHO}\right\rangle\left\langle S_0^{DHO}\right| + \left|S_0^{DHO}\right\rangle\left\langle S_1^{DHO}\right|\right) \tag{5}$$

4. Supplementary Figures

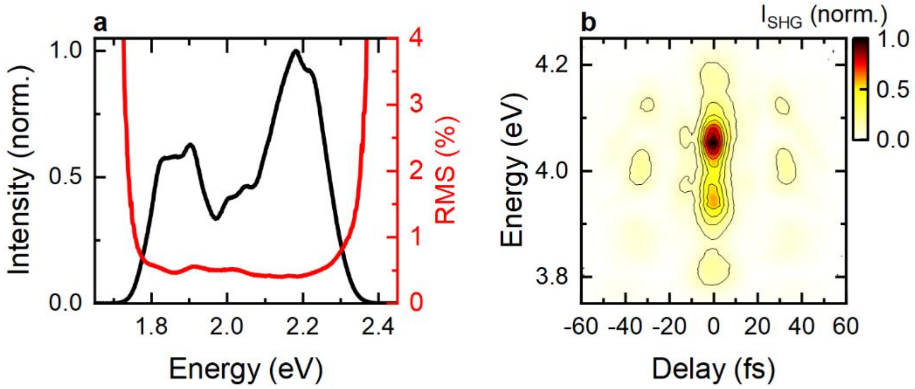

Figure S1. a, Normalized spectrum of the NOPA pulses used to excite and probe the A-D-A molecule. **b,** SHG-FROG map showing the normalized intensity of the SHG signal from the cross-correlation between the pump and probe beams in the 2DES setup at the sample position, from which a duration of ~9.3 fs is retrieved.

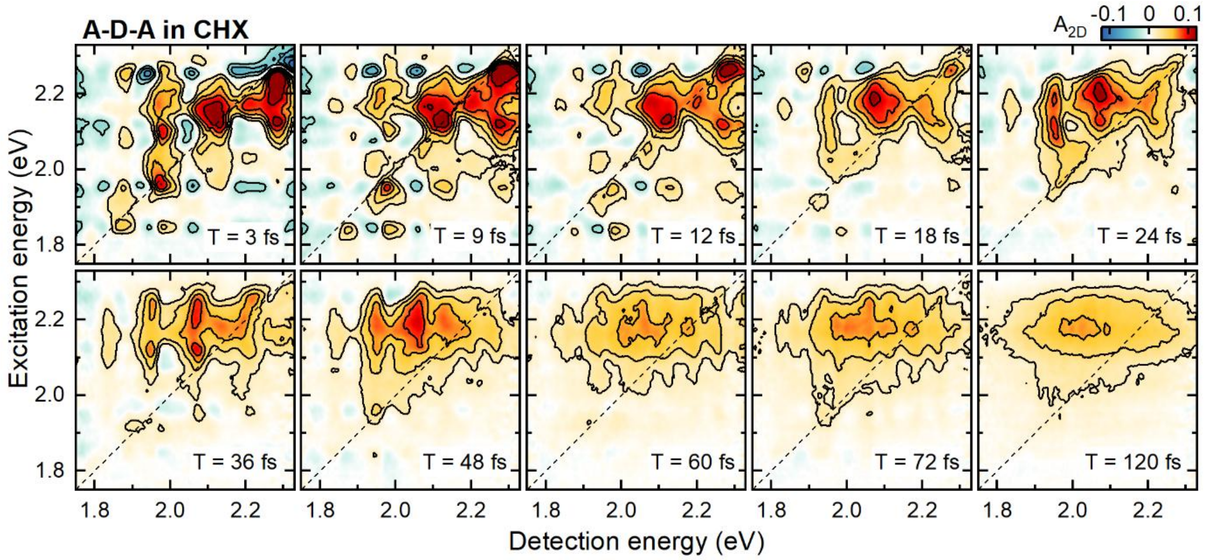

Figure S2. Absorptive 2DES maps of the A-D-A molecule in cyclohexane (CHX) for selected waiting times T up to 120 fs.

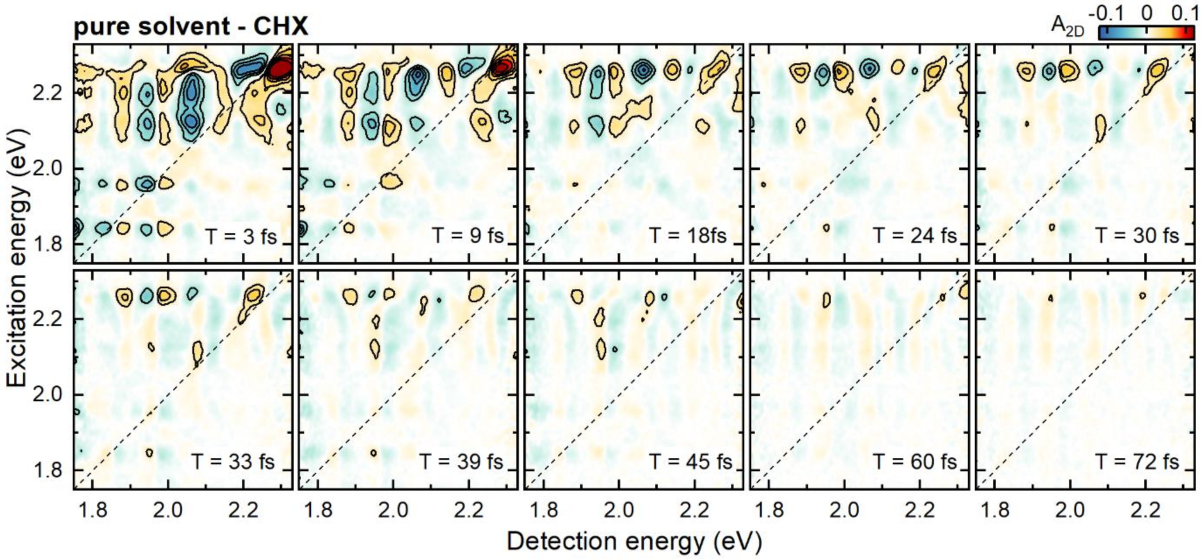

Figure S3. Absorptive 2DES maps of the pure cyclohexane (CHX) solvent for selected waiting times T showing that the off-resonant solvent response becomes negligible beyond ~18 fs.

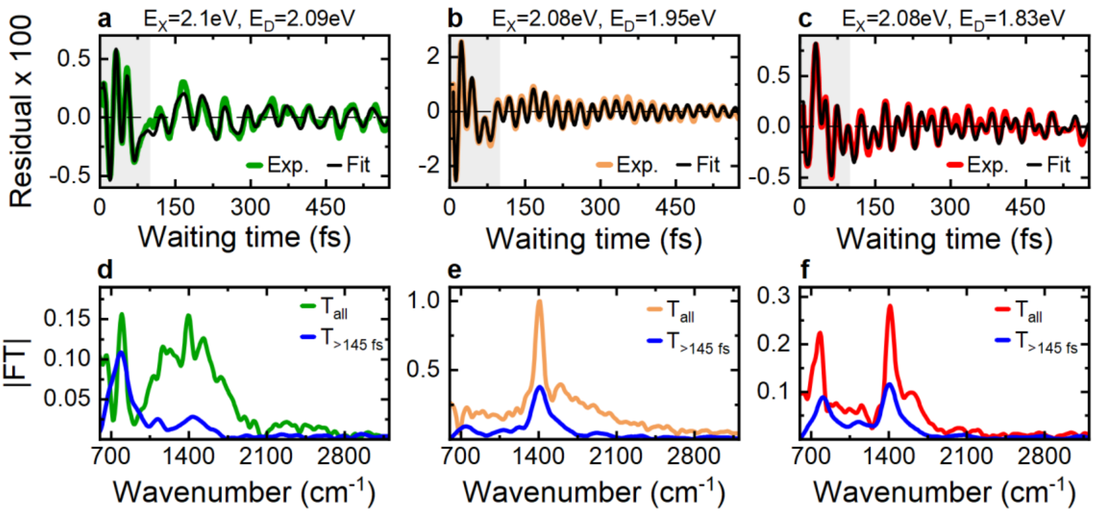

Figure S4. a,b,c, Residual amplitude oscillations at selected peaks in the experimental 2DES maps together with the corresponding time-domain fits (black curves). **d,e,f,** FT spectra of the corresponding residuals in (a,b,c) taken along the entire investigated waiting time window (T_{all}, green, orange, red) and for waiting times beyond 145 fs ($T_{>145\mathrm{fs}}$, blue).

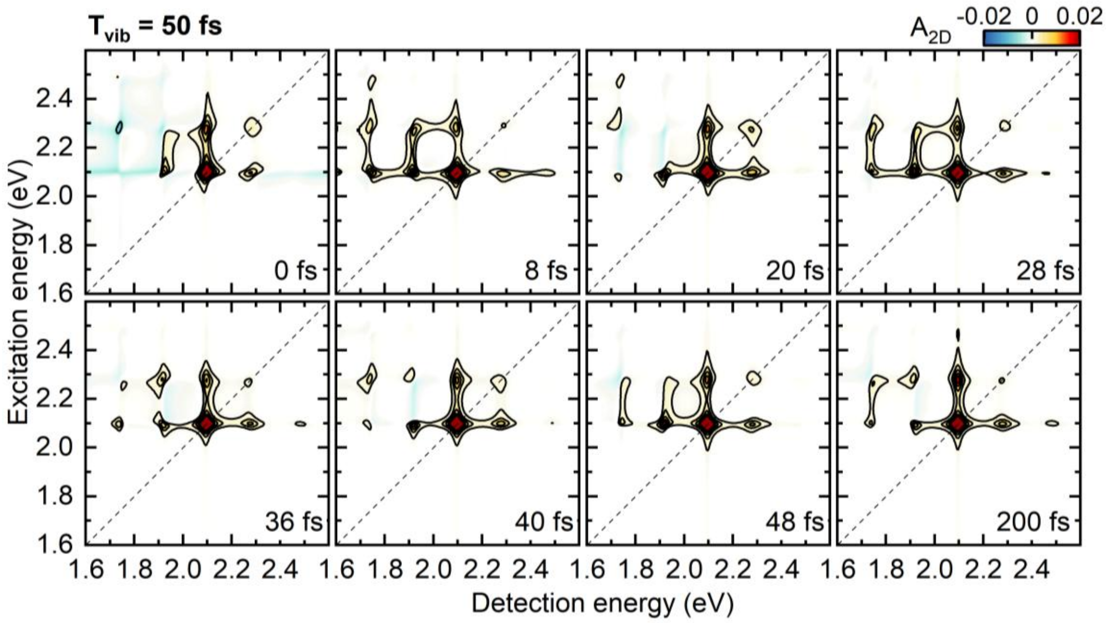

Figure S5. Simulated absorptive 2DES maps at selected waiting times T obtained using the ESM Hamiltonian.

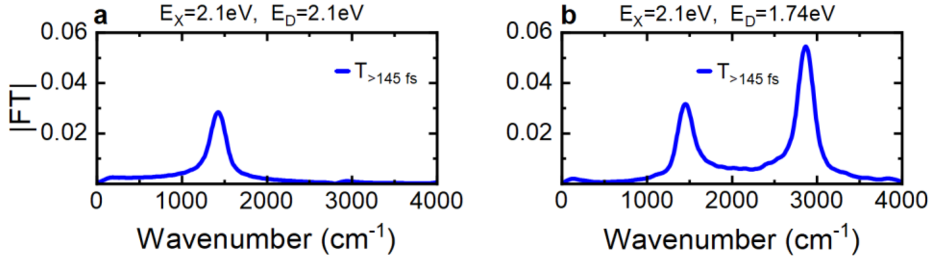

Figure S6. FT spectra of the long-lived oscillations taken from the simulated 2DES in Figure 2e for waiting times beyond 145 fs ($T_{>145\text{fs}}$). **a,b,** FT at (a) the diagonal peak at 2.1 eV and (b) the lowest energy cross-peak at E_X =2.1 eV, E_D = 1.74 eV show the 1430 cm^{-1} mode probing undamped vibrational wavepacket motion along Q_+ in the ground state. Additionally, in (b), the first overtone at ~2860 cm^{-1} is strongly enhanced due to the creation of a squeezed vibrational wavepacket along Q_-.

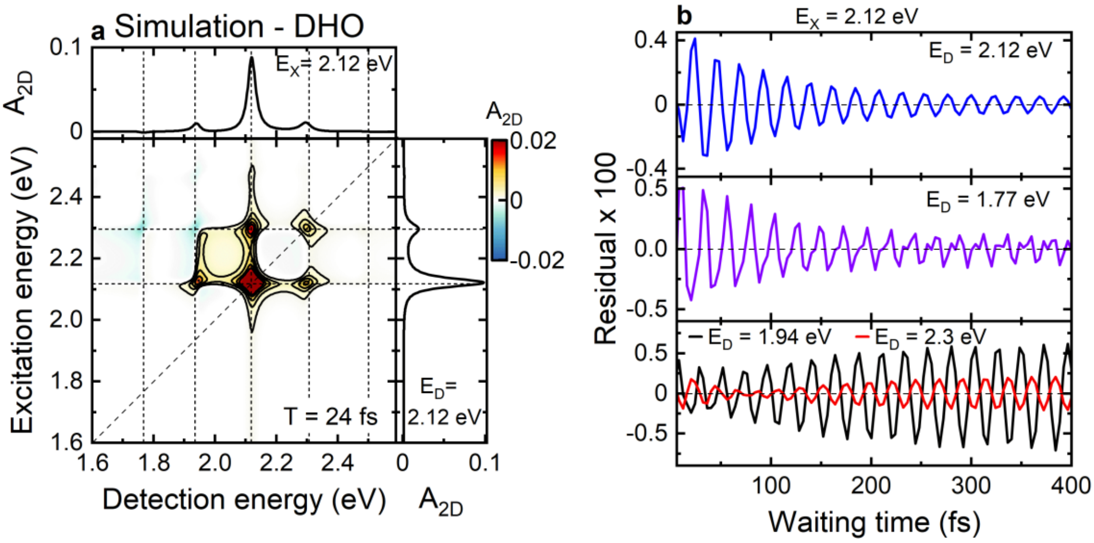

Figure S7. Simulated absorptive 2DES dynamics of a two-mode displaced harmonic oscillator (DHO) model using the same mode parameters as for the ESM. The displacement of the Q_+ mode with a frequency of 1430 cm^{-1} is 0.75, while the Q_- remains undisplaced. **a,** Exemplary simulated absorptive 2DES map at T = 24 fs showing a grid-like peak pattern with equally spaced peaks along both excitation and detection energy axes. The peak spacing of 178 meV is the energy of the Q_+ mode. The peak pattern is symmetric around the zero-phonon line resonance at 2.12 eV along the detection energy axis (inset, horizontal cross-section) in contrast to the ESM (cf. Figure 2a). **b,** Residual amplitude oscillations of selected peaks show persistent 23-fs oscillations arising from coherent vibrational wavepacket motion in the ground state and excited state. The dynamics of the residuals at the zero-phonon line diagonal peak (E_D = 2.12 eV, blue) and at the lowest-lying cross-peak (E_D = 1.77 eV, violet) feature a pronounced, initial ~100 fs decay arising from relaxation of the excited state wavepacket on this timescale, whereas the ground state vibrational motion is not damped.

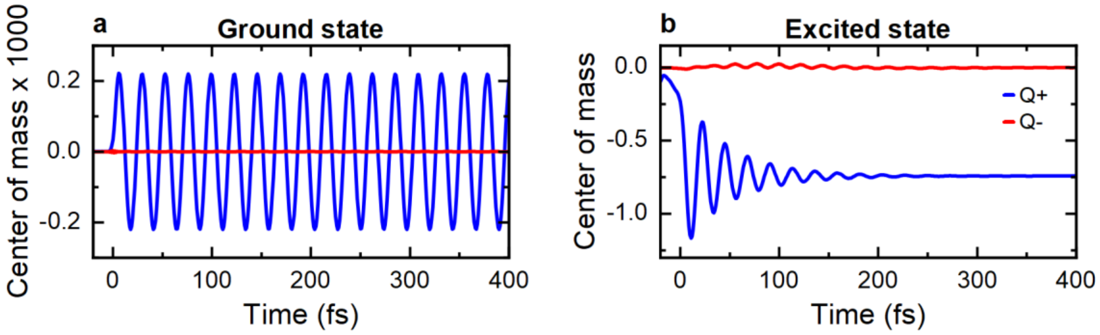

Figure S8. Dynamics of the center-of-mass motion of the optically launched coherent wavepacket in (a) the ground state and (b) the excited state along (blue) the symmetric Q_+ and (red) antisymmetric Q_- coordinates as obtained from ESM. In the excited state, vibrational relaxation with a time constant of 50 fs along both coordinates rapidly damps the oscillations, whereas in the ground state the wavepacket remains undamped. Importantly, the simulation shows that, (a) in the ground state, the wavepacket oscillates along Q_+, but not along Q_-. Moreover, the oscillation amplitude along Q_+ is three orders of magnitude weaker in the ground state compared to the excited state.

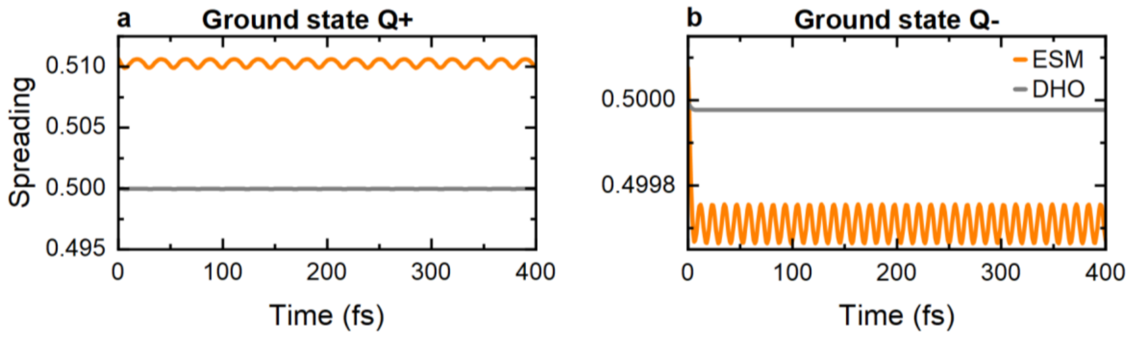

Figure S9. a,b, Dynamics of the wavepacket spreading in the ground state S_0 along (a) the symmetric Q_+ and (b) antisymmetric Q_- coordinates obtained by calculating the second central moment of each coordinate. Results from ESM (orange) are compared to those of a DHO model (gray) using the same displacement parameters. The DHO wavepacket (gray) shows a time independent spreading of 0.5 as expected for a classical wavepacket motion, consistent with a coherent state. The ESM simulations in contrast reveal "breathing" of the ground state wavepacket (orange), mainly around Q_-, with ~11-fs period, half that of the vibrational mode. This suggests the excitation of a non-classical, squeezed ground state wavepacket along Q_-.

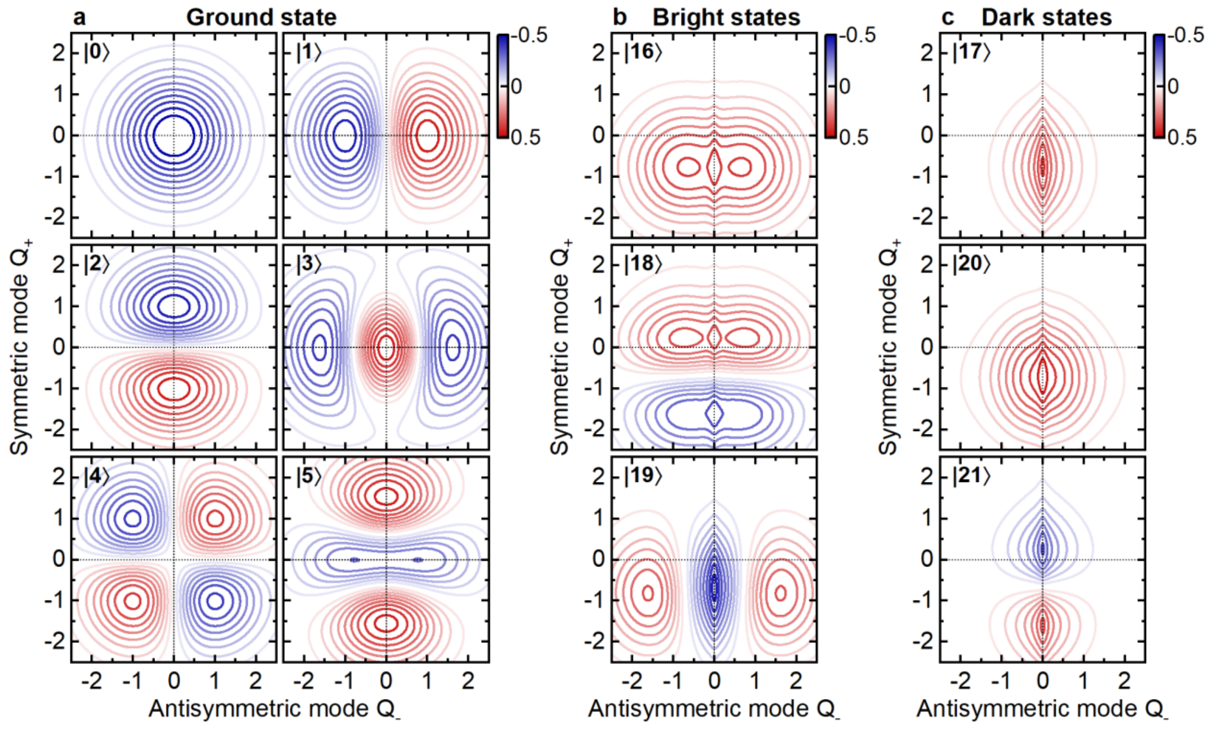

Figure S10. Calculated amplitude of the wavefunctions of selected vibronic eigenstates in the effective coordinate plane along the symmetric and antisymmetric mode.

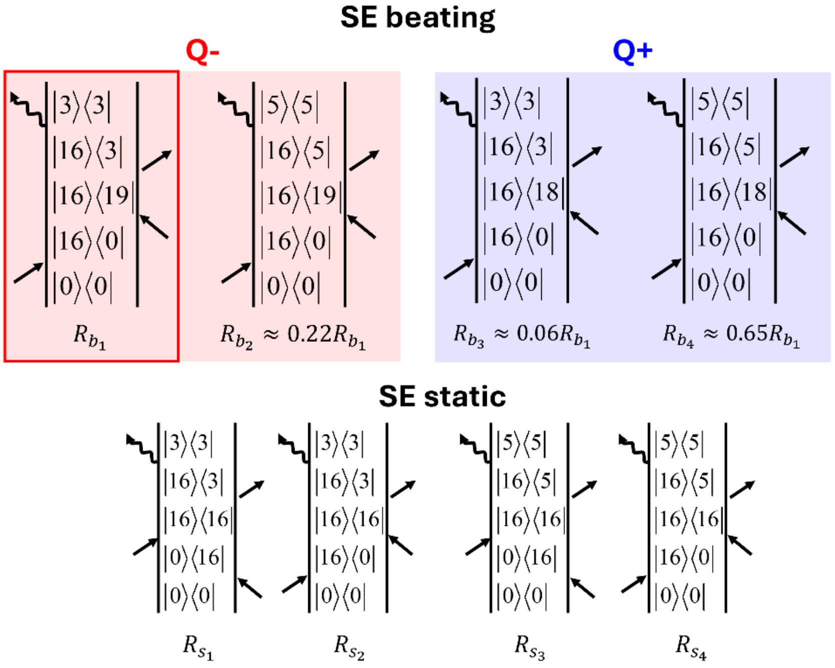

Figure S11. Double sided Feynman diagrams describing stimulated emission (SE) pathways leading to the emergence of the lowest energy cross-peak at $E_X = E_0, E_D = E_0 - 2\hbar\omega_v$ in the 2DES maps. The upper row shows SE pathways that contribute an oscillating peak amplitude with period T_v as a function of the waiting time. They probe excited state wavepacket motion along (shaded red) Q_- and (shaded blue) Q_+. The dominant contribution to the peak amplitude oscillations ($R_{b_1} \propto \mu_{16,0}\mu_{19,0}\mu_{3,19}\mu_{3,16}$) is highlighted by a red frame. It arises from the motion along Q_-. The relative contribution of the other pathways to the amplitude oscillations is indicated below each diagram. The lower row reports static SE pathways at the same peak position reflecting population dynamics in $|16\rangle$ as a function of the waiting time.

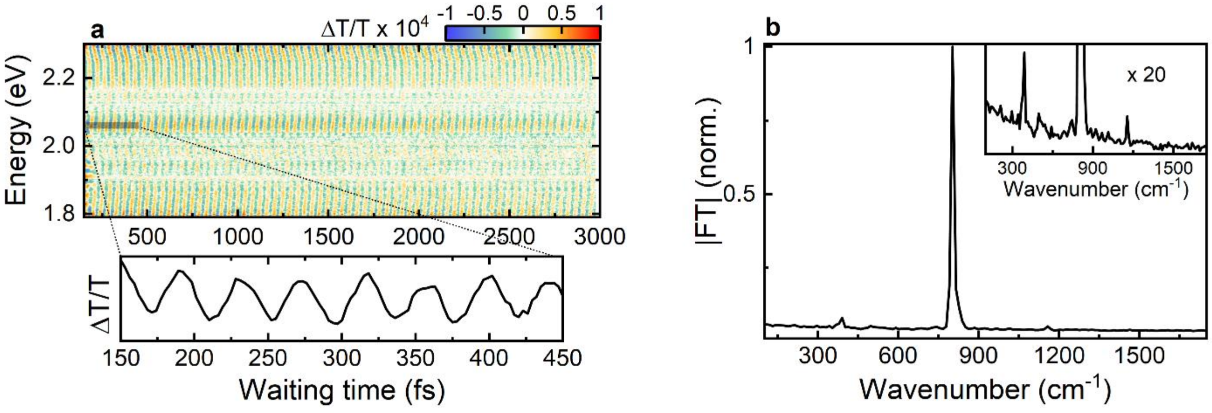

Figure S12. **a**, Differential transmission $\Delta T/T$ map of the pure solvent cyclohexane as a function of the probe energy for waiting times up to 3 ps. The map shows temporal oscillations over the entire probe energy range and persisting beyond our investigated waiting time window of 3 ps. The dominant period of the oscillations is of ~40 fs (inset). **b**, Fourier transform (FT) amplitude of the map in (a) integrated along the probe energy axis confirms a strong, sharp frequency component at 803 cm^{-1} (~40 fs), corresponding to the dominant Raman mode of cyclohexane. Additionally faint modes are also observed at ~390 cm^{-1}, 496 cm^{-1} and 1156 cm^{-1}. The FT amplitude of these modes is more than 20 times weaker than that of the 800 cm^{-1} mode, thus indicating that the presence of these additional modes does not influence the intramolecular dynamics observed in Figure 2 and Figure S4.

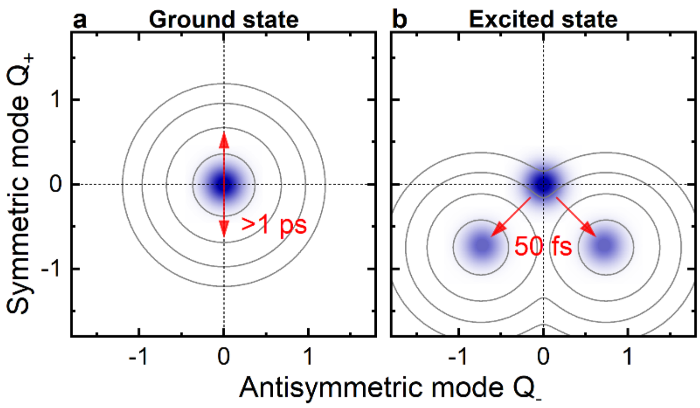

Figure S13. Schematic illustration of the relevant dynamical processes in the ground and excited state. Contour lines show the effective potential energy surface of the (a) ground and (b) excited electronic states as a function of the symmetric Q_+ and antisymmetric Q_- coordinates. The blue areas schematically depict the nuclear wavepacket. **a**, Coherent wavepacket motion in the ground state persists for > 1 ps oscillating mainly along Q_+. **b**, Excited state wavepacket motion in contrast proceeds as a splitting and refocusing along Q_- until it relaxes in the two equivalent minima with a decay time of ~50 fs.

5. Supplementary Tables

Table S1. Calculated transition dipole moment (TDM) matrix elements associated with absorption transitions from the ground state $|0\rangle$ to the excited state vibronic manifold $|j\rangle$, with $j \geq 16$.

Eigenstate $\lvert j\rangle$	Eigenenergy (eV)	TDM from $\lvert 0\rangle$ to excited state $\mu_{j,0} = \langle j\lvert\hat{\mu}_S\rvert 0\rangle$	Eigenstate $\lvert j\rangle$	Eigenenergy (eV)	TDM from $\lvert 0\rangle$ to excited state $\mu_{j,0} = \langle j\lvert\hat{\mu}_S\rvert 0\rangle$
16	2.096	7.81e-2	32	2.787	-1.55e-3
17	2.108	-1.09e-16	33	2.789	-8.96e-17
18	2.278	3.61e-2	34	2.798	-1.49e-3
19	2.284	-3.66e-2	35	2.798	-6.72e-17
20	2.285	-2.77e-15	36	2.886	-1.11e-16
21	2.29	-1.08e-15	37	2.89	1.82e-3
22	2.466	-1.69e-2	38	2.971	-8.34e-17
23	2.467	-3.84e-15	39	2.975	6.98e-4
24	2.489	1.2e-2	40	2.976	4.41e-16
25	2.493	-8.62e-17	41	2.98	-7.44e-4
26	2.497	-1.17e-2	42	3.182	5.76e-17
27	2.501	2.28e-16	43	3.184	-6.69e-17
28	2.675	-8.02e-16	44	3.188	2.11e-4
29	2.677	5.8e-3	45	3.191	-2.6e-4
30	2.678	-6.99e-16	46	3.481	8.9e-17
31	2.679	-5.32e-3	47	3.488	3.13e-05

Table S2. Calculated transition dipole moment (TDM) matrix elements associated with stimulated emission transitions from three selected bright excited vibronic states to the ground state manifold $|j\rangle$, with $j < 16$.

**Eigenstate** $|j\rangle$	**TDM from excited state** $|16\rangle$ **to ground** $\mu_{j,16} = \langle j|\hat{\mu}_S|16\rangle$	**TDM from excited state** $|18\rangle$ **to ground** $\mu_{j,18} = \langle j|\hat{\mu}_S|18\rangle$	**TDM from excited state** $|19\rangle$ **to ground** $\mu_{j,19} = \langle j|\hat{\mu}_S|19\rangle$
0	7.82e-2	3.61e-2	-3.66e-2
1	1.45e-13	-1.94e-13	-4.7e-14
2	4.47e-2	5.99e-2	1.63e-2
3	2.09e-2	-3.12e-3	5.21e-2
4	1.86e-15	-6.66e-14	-6.11e-14
5	-1.31e-2	5.51e-2	1.87e-2
6	9.82e-3	-1.12e-2	3.26e-2
7	-8.97e-17	-2.012e-16	-7.93e-16
8	-4.08e-17	-4.92e-16	-2.23e-15
9	-5.42e-3	2.36e-2	1.39e-3
10	4.02e-3	-1.11e-2	1.36e-2
11	1.57e-17	5.04e-16	1.30e-15
12	-3.33e-19	1.23e-16	8.16e-17
13	2.57e-17	-3.51e-16	-3.87e-16
14	-1.26e-3	5.06e-3	-4.29e-2
15	-6.35e-19	-7.78e-17	-1.47e-16

Table S3. Frequency components and their decay times as obtained from the analysis of the residual oscillations in Figure 2b and Figure S4 for each trace on the entire investigated waiting time window.

a) Fitting parameters corresponding to the residual trace in Figure S4a

**Frequency (cm^{-1})**	**Decay time (fs)**	**Amplitude**	**Phase (rad)**
200	160	0.0029	6.028
653	109	0.0034	6.133
795	978	0.0013	0.323
1146	39	0.0087	6.283
1405	395	0.0005	5.092
1620	40	0.0075	2.068

b) Fitting parameters corresponding to the residual trace in Figure S4b

Frequency (cm^{-1})	Decay time (fs)	Amplitude	Phase (rad)
200	187	0.0052	6.283
795	500	0.0001	6.283
1405	398	0.0076	6.283
1572	128	0.0031	6.283
1678	40	0.0186	5.363

c) Fitting parameters corresponding to the residual trace in Figure S4c

Frequency (cm^{-1})	Decay time (fs)	Amplitude	Phase (rad)
160	90	0.0062	0.344
795	1353	0.0010	0.074
810	51	0.0060	0.863
1404	397	0.0024	5.427
1678	50	0.0036	1.7845

6. References

1 Winte, K. *et al.* Vibronic coupling-driven symmetry breaking and solvation in the photoexcited dynamics of quadrupolar dyes. *Nature Chemistry* **17**, 1742–1749 (2025).

2 Quenzel, T. *et al.* Plasmon-Enhanced Exciton Delocalization in Squaraine-Type Molecular Aggregates. *ACS Nano* **16**, 4693-4704 (2022).

3 Souri, S. *et al.* Ultrafast Time-Domain Spectroscopy Reveals Coherent Vibronic Couplings upon Electronic Excitation in Crystalline Organic Thin Films. *Journal of Physical Chemistry Letters* **15**, 11170-11181 (2024).

4 Brida, D., Manzoni, C. & Cerullo, G. Phase-locked pulses for two-dimensional spectroscopy by a birefringent delay line. *Opt. Lett.* **37**, 3027-3029 (2012).

5 Réhault, J., Maiuri, M., Oriana, A. & Cerullo, G. Two-dimensional electronic spectroscopy with birefringent wedges. *Review of Scientific Instruments* **85**, 123107 (2014).

6 Terenziani, F., Painelli, A., Katan, C., Charlot, M. & Blanchard-Desce, M. Charge Instability in Quadrupolar Chromophores: Symmetry Breaking and Solvatochromism. *Journal of the American Chemical Society* **128**, 15742-15755 (2006).

7 Sissa, C., Delchiaro, F., Di Maiolo, F., Terenziani, F. & Painelli, A. Vibrational coherences in charge-transfer dyes: a non-adiabatic picture. *J Chem Phys* **141**, 164317 (2014).

8 Ivanov, A. I. Symmetry breaking charge transfer in excited multibranched molecules and dimers: A unified standpoint. *Journal of Photochemistry and Photobiology C: Photochemistry Reviews* **58**, 100651 (2024).

9 Zheng, C., Zhong, C., Collison, C. J. & Spano, F. C. Non-Kasha Behavior in Quadrupolar Dye Aggregates: The Red-Shifted H-Aggregate. *The Journal of Physical Chemistry C* **123**, 3203-3215 (2019).

10 Zhong, C., Bialas, D., Collison, C. J. & Spano, F. C. Davydov Splitting in Squaraine Dimers. *The Journal of Physical Chemistry C* **123**, 18734-18745 (2019).

11 De Sio, A., Nguyen, X. T. & Lienau, C. Signatures of Strong Vibronic Coupling Mediating Coherent Charge Transfer in Two-Dimensional Electronic Spectroscopy. *Zeitschrift für Naturforschung A* **74**, 721-737 (2019).
12 Seidner, L., Stock, G. & Domcke, W. Nonperturbative approach to femtosecond spectroscopy: General theory and application to multidimensional nonadiabatic photoisomerization processes. *The Journal of Chemical Physics* **103**, 3998-4011 (1995).
13 Fresch, E. *et al.* Two-dimensional electronic spectroscopy. *Nature Reviews Methods Primers* **3**, 84 (2023).
